# Predictive Value of Preoperative High-Sensitive C-reactive Protein (hs-CRP)/Albumin Ratio in Systemic Inflammatory Response Syndrome (SIRS) After Semi-rigid Ureteroscopy

**DOI:** 10.7759/cureus.23117

**Published:** 2022-03-13

**Authors:** Musab A Kutluhan, Selman Unal, Asim Ozayar, Emrah Okulu, Onder Kayigil

**Affiliations:** 1 Urology, Ankara Yildirim Beyazit University School of Medicine, Ankara, TUR

**Keywords:** urinary tract stones, infection, ureteroscopy, systemic inflammatory response syndrome, high-sensitive c-reactive protein

## Abstract

Objective: To determine the predictive value of high-sensitive C-reactive protein (hs-CRP)/albumin ratio in systemic inflammatory response syndrome (SIRS) after semi-rigid ureteroscopy (URS).

Material and Methods: Between April 2021 and October 2021, 148 patients who had ureteral stone treatment with a ureteroscope in our hospital were included. Preoperative hs-CRP/albumin ratio was obtained by dividing the hs-CRP level by the albumin level. High-sensitivity modified Glasgow prognostic score (hs-mGPS) was obtained according to hs-CRP and albumin values. Two groups were identified as post-URS SIRS positive and negative. Inflammation biomarkers were evaluated in groups.

Results: There was a statistically significant difference between groups in terms of preoperative hs-CRP, albumin, and hs-CRP/albumin ratio (p < 0.001, p = 0.003, and p < 0.001, respectively). The optimal cutoff value for the hs-CRP/albumin ratio was 0.04651. While the risk of developing SIRS after surgery was 72.73% in patients with a hs-CRP/albumin ratio higher than 0.04651, the chance of not developing SIRS was 87.5% in patients below this value. The probability of developing SIRS was found to be significantly different in hs-mGPS (p < 0.001).

Conclusion: Our study indicated that hs-CRP/albumin ratio can predict post-URS SIRS. Larger-scale, multicentric prospective studies should certainly be done to validate the predictive value of hs-CRP/albumin ratio in post-URS SIRS.

## Introduction

Semi-rigid ureteroscopy (URS) is a method recommended in current urology guidelines in the treatment of ureteral stones [1]. Urinary tract infection could occur after URS [2]. The rate of infectious complications after URS is 9%-25% [3]. Infectious complications range from asymptomatic bacteriuria to septic shock. It is of great importance to predict infectious complications after URS.

In patients who were operated after a negative urine culture, it has been reported that various factors can cause post-URS fever [4]. Various preoperative serum markers, such as neutrophil-to-lymphocyte ratio (NLR) and lymphocyte-to-monocyte ratio (LMR), among others, can predict systemic inflammatory response. The predictive value of these markers for postoperative systemic inflammatory response syndrome (SIRS) after kidney stone surgery was shown in some studies. NLR and LMR have been reported to be effective in predicting SIRS after percutaneous nephrolithotomy (PNL) [5]. High-sensitive C-reactive protein (hs-CRP)/albumin ratio is a novel inflammation marker. Studies indicating the predictive value of the hs-CRP/albumin ratio in SIRS after endoscopic stone surgery are limited. The high-sensitivity modified Glasgow prognostic score (hs-mGPS), obtained according to hs-CRP and albumin levels, is a new systemic inflammation biomarker that provides sensitive measurements due to the use of lower threshold values. Studies have shown that hs-mGPS is a better prognostic factor than mGPS [6,7]. The predictive value of hs-CRP/albumin in post-PNL SIRS has been demonstrated [8]. The role of hs-CRP/albumin ratio in predicting SIRS after URS is still unclear. In this study, our purpose is to determine the predictive value of hs-CRP/albumin ratio in post-URS SIRS.

## Materials and methods

Patient selection

After local ethics committee approval (26379996/133), between April 2021 and October 2021, 148 patients who had ureteral stone treatment with a 9.5 Fr semi-rigid ureteroscope (Karl Storz, Tuttlingen, Germany) were included in our prospective observational study. Age, gender, body-mass index, comorbidities, and operation times were recorded. Preoperative direct urinary system radiography and noncontrast abdominal tomography were performed in all patients. Stone size, localization, and side were recorded according to preoperative imaging. Preoperative albumin, hs-CRP values (within 1 week) were recorded. Preoperative urinalysis and urine culture were studied in all patients. The leukocyte counts in the preoperative urinalysis were recorded. All patients received prophylactic 1 g cefazolin intravenously 30 minutes before surgery as a routine procedure. After lithotripsy, 22-26 cm 4.8 F double j stent (GEOTEK, Ankara, Turkey) was used according to the height index. Postoperative body temperature, heart rate, blood pressure, and respiratory rates of the patients were followed up on. Patients were divided into two groups: post-URS SIRS positive and negative.

Inflammation biomarkers

The hs-CRP/albumin ratio was obtained by dividing the hs-CRP level by the albumin level. hs-mGPS was obtained according to the hs-CRP and albumin values. Patients with an albumin level of <35 g/L and hs-CRP level of >3 mg/L were given a score of 2. Patients with an albumin level of >35 g/L and hs-CRP level of >3 mg/L were given a score of 1. The remaining patients were assigned a score of 0 [9]. The groups were compared in terms of inflammation biomarkers (hs-CRP/albumin ratio and hs-mGPS).

Inclusion and exclusion criteria

Patients who underwent unilateral semi-rigid URS due to ureteral stone were included in our study. Patients who have undergone bilateral endoscopic intervention, whose preoperative urine culture was not sterile, who used preoperative antibiotics for urinary tract infection, who had postoperative residual stones, who developed SIRS or urosepsis due to urinary system stone disease, who had percutaneous nephrostomy or double j catheter for treatment of acute pyelonephritis, whose operation was terminated due to the detection of intraoperative pyuria, and who had a known hematological disease, solid organ cancer, and a history of renal failure were not included in the study. Patients who used immunosuppressive therapy and chemotherapy were also excluded.

Endpoint

The occurrence of SIRS after URS was the endpoint of this study. SIRS was identified by two or more of these criteria: (1) body temperature >38°C or <36°C, (2) heart rate > 90 bpm, (3) respiratory rate > 20 breaths/min or PaCO2 < 32 mm Hg, and (4) white blood cell count > 12 × 109 or 4 × 109 cells/L [10].

Statistical analyses

SPSS 23.0 software was used for data analyses (SPSS, Version 23.0; IBM Corp, Armonk, NY). Mann-Whitney U test for continuous variables and Fisher’s Exact and Chi-Square tests for categorical variables were applied to analyze the differences between groups. The optimal cutoff value of the hs-CRP/albumin ratio was identified by receiver operating characteristic (ROC) analysis. p < 0.05 was evaluated as statistically significant.

## Results

SIRS developed in 16 (10.9%) of the patients. The mean ages of patients with and without SIRS were 50.06 ± 16.20 years and 44.69 ± 11.71 years, respectively. Patients who developed SIRS were treated with appropriate antibiotics by the infectious diseases clinic. Descriptive features of patients are shown in Table 1.

**Table 1:** Descriptive features of patients SIRS: systemic inflammatory response syndrome, BMI: body-mass index, SD: standard deviation

	No SIRS	SIRS
Number of patients, N	132	16
Gender, N (%)		
Male	92 (69,7%)	8 (50%)
Female	40 (30,3%)	8 (50%)
Age median (Mean ± SD), years	44,69 ± 11,71	50,06 ± 16,20
BMI median (Mean ± SD), kg/m^2^	26,99 ± 4,21	27,41 ± 3,58
Comorbidities, N (%)		
None	102	6
Hypertension	13	4
Diabetes mellitus	19	6
Coronary heart disease	1	0
Cerebrovascular disease	0	0
Stone location, N (%)		
Distal ureter	47 (35,6%)	7 (43,75%)
Mid ureter	40 (30,3%)	3 (18,75%)
Proximal ureter	45 (34,09%)	6 (37,5%)
Stone size median (Mean ± SD), cm	0,93 ± 0,30	0,88 ± 0,26
Stone side, N (%)		
Right	76 (57,57%)	10 (62,5%)
Left	56 (42,42%)	6 (37,5%)
Operation time (Mean ± SD), minute	30 ± 5,5	33 ± 4,7

The hs-CRP value in the group with SIRS was statistically significantly higher than in the group without SIRS (p < 0.001). Also, albumin value in the group with SIRS was statistically significantly higher than in the group without SIRS (p = 0.003). In addition, the hs-CRP/albumin ratio was statistically significantly higher in the group with SIRS than in the group without SIRS (p < 0.001) (Table 2).

**Table 2:** Comparison of groups according to hs-CR, albumin, hs-CRP/albumin ratio, and hs-mGPS hs-CRP: high-sensitive C-reactive protein, SIRS: systemic inflammatory response syndrome, hs-mGPS: high-sensitivity modified Glasgow prognostic score, IQR: interquartile range (Mann-Whitney U and chi-square statistical tests were applied)

	No SIRS, 132 (89,1%)	SIRS, 16 (10,9%)	P value
hs-CRP (mg/L) Median (IQR)	1,10 (0,60–1,10)	4,05 (2,43–14,60)	<0.001
Albumin (g/L) Median (IQR)	44,00 (41,00–47,00)	40,00 (33,00–45,00)	0.003
hs-CRP/Albumin Median (IQR)	0,02 (0,01–0,05)	0,10 (0,06–0,37)	<0.001
hs-mGPS N (%)			
0	109 (82,57%)	4 (25%)	
1	22 (16,66%)	6 (37,5%)	
2	1 (0,75%)	6 (37,5%)	<0.001

**Figure 1:**
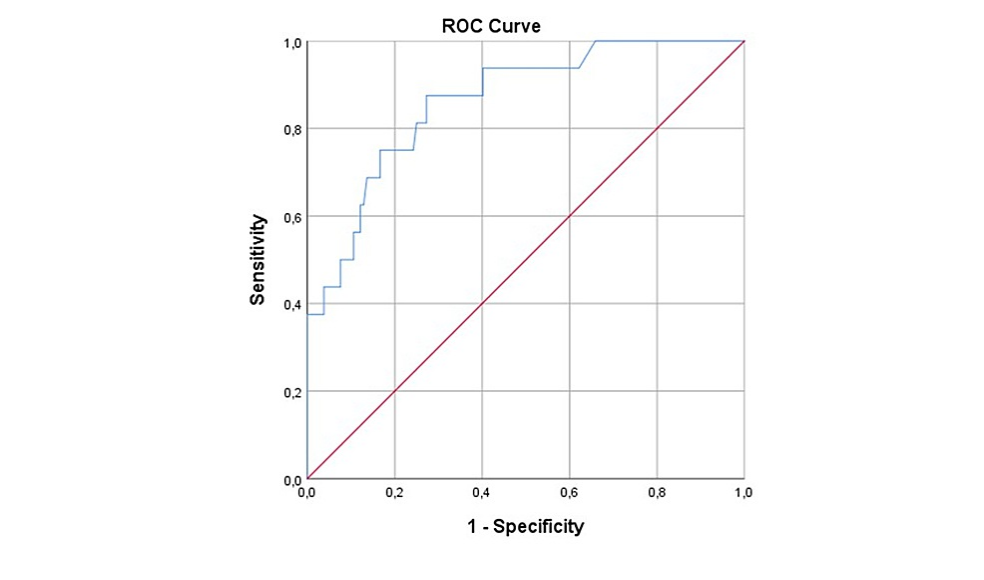
ROC analyses (hs-CRP/albumin ratio) in the prediction of post-URS SIRS Youden index J: 0.6023, associated criterion >0.046511628, sensitivity: 87.50, specificity: 72.73 ROC: receiver operating characteristic, hs-CRP: high-sensitive C-reactive protein, URS: ureteroscopy, SIRS: systemic inflammatory response syndrome

According to ROC analysis, the optimal cutoff value for the hs-CRP/albumin ratio was 0.04651. While the risk of developing SIRS after surgery was 72.73% in patients with a hs-CRP/albumin ratio higher than 0.04651, the chance of not developing SIRS was 87.5% in patients with a hs-CRP/albumin ratio below this value (Figure 1).

With regard to hs-mGPS, 109 (82.57%) of the patients in the group without SIRS had a score of 0, while four (25%) of patients with SIRS had a score of 0. In addition, six (37.5%) patients in the group with SIRS had a score of 2, while one (0.75%) patient without SIRS had a score of 2. The rate of not developing SIRS was statistically significantly higher in those with hs-mGPS 0 (p < 0.001). In addition, the probability of developing SIRS was shown to be significantly higher in those with hs-mGPS 2 (p < 0.001). As a conclusion, the probability of developing SIRS was found to be significantly different in hs-mGPS, and the probability of developing SIRS increased as hs-mGPS increased (Table 2). 

## Discussion

Till date, various predictive factors for post-URS infectious complications have been demonstrated in studies. In a large population study, Southern et al. showed that positive preoperative urine culture, prolonged surgery time, and higher Charlson comorbidity index were important markers for post-URS SIRS [11]. In another study by Mitsuzuka et al., it was stated that preoperative pyuria and acute pyelonephritis were important factors for post-URS fever [12]. In addition, various comorbidities such as diabetes mellitus have been shown to be a risk factor for post-URS fever [13]. In clinical practice, post-URS fever and SIRS can also be seen in patients with a clean urine culture and no history of pyelonephritis. The predictive value of preoperative serum markers (NLR, platelet-to-lymphocyte ratio (PLR), etc.) in postoperative SIRS was demonstrated in various studies. Tang et al. indicated that preoperative NLR had a predictive value in post-PNL SIRS [5]. In other study by Akdeniz et al., it was stated that preoperative PLR was an independent risk factor for post-PNL [14].

To best of our knowledge, our study is the first to show that the preoperative hs-CRP/albumin ratio can predict post-URS SIRS. In the literature, there is only one study evaluating the predictive value of the hs-CRP/albumin ratio for postoperative SIRS in patients operated for kidney stone disease [8]. In one study, it was shown that the hs-CRP/albumin ratio could predict post-PNL SIRS [8]. It stated that a significant difference was obtained in terms of hs-CRP/albumin ratio between the groups with and without post-PNL SIRS (0.17 (0.06-0.35) versus 0.03 (0.02-0.06), respectively, p < 0.001). Also, they stated that the optimal cutoff value of the hs-CRP/albumin ratio was 0.06. In our study, we found a difference between the groups regarding hs-CRP/albumin ratio (0.10 (0.06-0.37) versus 0.02 (0.01-0.05), respectively, p < 0.001). In addition, the optimal cutoff value of the hs-CRP/albumin ratio was 0.047. Our findings are identical to the literature.

As a reactant, CRP increases secondary to inflammation [15]. An obstructing ureteral stone can cause inflammation, and CRP levels increase. In a study by Barut et al., CRP levels were higher in patients with ureteral stones who had acute pyelonephritis compared with patients with lower urinary tract infections [16]. Yamamichi et al. stated that an increase in CRP level was an independent factor in patients with acute pyelonephritis due to proximal obstructing ureteral stones [17]. In another study by Angulo et al., it was shown that high CRP values were more significant than leukocytosis for the decision of drainage in patients with obstructive ureteral stones and renal colic [18]. Studies in the literature show that CRP level is an important marker for acute pyelonephritis in patients with ureteral stones. In our study, the hs-CRP level in patients who developed post-URS SIRS was higher than in patients who did not develop SIRS (p < 0.001). The results of our study indicate that an increase in hs-CRP level due to ureteral stones may be a risk factor for post-URS SIRS.

Inflammation can decrease serum albumin levels. Although this decrease is attributed to various factors, the increase in protein catabolism and decrease in hepatic synthesis are known as the main factors [19]. Low serum albumin levels increase the risk of postsurgical infectious complications [20,21]. In addition, it was demonstrated that preoperative serum albumin values in patients with postflexible URS sepsis are significantly lower than in patients without sepsis [22]. In our study, preoperative serum albumin values in patients with post-URS SIRS were lower than in patients without SIRS (p = 0.003).

Another marker indicating systemic inflammation is mGPS. mGPS has been shown to have prognostic value in various urological cancers [23,24]. Also, hs-mGPS was shown to be a predictive marker for post-PNL SIRS [10]. In our study, the probability of developing SIRS was found to be significantly different in hs-mGPS.

Nevertheless, our study has some limitations. First, our study is a single-center study and the study population was limited. Second, we did not perform stone analyses on all patients. Therefore, stone type could affect inflammation markers as well as the occurrence of SIRS. Third, we did not compare hs-CRP/albumin ratio with other inflammation markers such as NLR. Lastly, hs-CRP could be affected by some situations other than inflammation.

## Conclusions

In conclusion, our study indicated that hs-CRP/albumin ratio can predict post-URS SIRS. We believe that patients with a high hs-CRP/albumin ratio should be monitored for the occurrence of post-URS SIRS. However, larger-scale, multicentric prospective studies should certainly be done to validate the predictive value of hs-CRP/albumin ratio in post-URS SIRS.
